# Balancing sample sizes in parasitology: A standardized experimental infection method using faecal parasite eggs and aquatic intermediate hosts

**DOI:** 10.1016/j.crpvbd.2026.100364

**Published:** 2026-03-06

**Authors:** Sara M. Rodríguez, Katherine Burgos-Andrade, Valentina Escares-Aguilera, Bárbara Gutiérrez, Nelson Valdivia

**Affiliations:** a Departamento de Ecología, Facultad de Ciencias, Universidad Católica de la Santísima Concepción, Concepción, Chile; b Centro de Investigación en Recursos Naturales y Sustentabilidad (CIRENYS), Universidad Bernardo O’Higgins, Avenida Viel 1497, Santiago de Chile, Chile; c Instituto de Ciencias Marinas y Limnológicas, Facultad de Ciencias, Universidad Austral de Chile, Valdivia, Chile; d Centro FONDAP Investigación en Dinámica de Ecosistemas Marinos de Altas Latitudes (IDEAL), Valdivia, Chile

**Keywords:** Acanthocephalans, Balancing samples, Experimental infection, Faecal egg count, Intermediate hosts, Seagulls

## Abstract

Balancing sample sizes between infected and uninfected hosts is a major challenge in ecological parasitology, particularly for intermediate hosts in complex life cycles. In natural populations, these hosts often accumulate parasites as they grow, making it difficult to obtain comparable numbers of infected and uninfected individuals across size classes. Here, we present a standardized and repeatable protocol to experimentally infect and re-infect intermediate hosts using parasite eggs obtained directly from fresh faeces of definitive hosts. We evaluated this approach in a marine host-parasite system involving the acanthocephalan *Profilicollis altmani*, the mole crab *Emerita analoga*, and the grey gull *Leucophaeus modestus*. Crabs were reared under controlled laboratory conditions from early juvenile stages to ensure they were parasite-free and were exposed to eggs through filtered faecal suspensions. Larval development was monitored across two inoculation events. The first larval stage (acanthellae) appeared six days post-inoculation and matured into cystacanths during the following week. After the first exposure, 83% (small) and 96% (large) of crabs were infected; following the second exposure, infection prevalence reached 100%. Larger crabs acquired more parasites than smaller individuals, likely due to higher filtration rates. This method closely mimics natural faecal-oral transmission and avoids the need to isolate or manipulate parasite eggs directly. It also addresses the common issue of parasite overdispersion in natural populations by enabling controlled infections across host size classes and experimental replicates. The technique is especially useful for species with opaque cuticle, where parasites cannot be visually detected *in vivo*, and can be potentially applied to other helminths (e.g. nematodes, cestodes, digeneans) whose eggs are shed in vertebrate faeces. Overall, this protocol provides an ecologically relevant and reliable approach for experimental parasitology in marine and freshwater systems.

## Highlights

• A standardized protocol to infect and re-infect intermediate hosts using eggs in faecal samples.
• Tested in the *Profilicollis* - *Emerita* - *Leucophaeus* system under controlled conditions.
• Two inoculations produced acanthellae by Day 6 and reached 100% infection prevalence.
• Larger crabs acquired more parasites, likely due to higher filtration rates.
• The method mimics natural faecal-oral transmission and avoids direct egg manipulation.

## Graphical abstract

**Figure:**
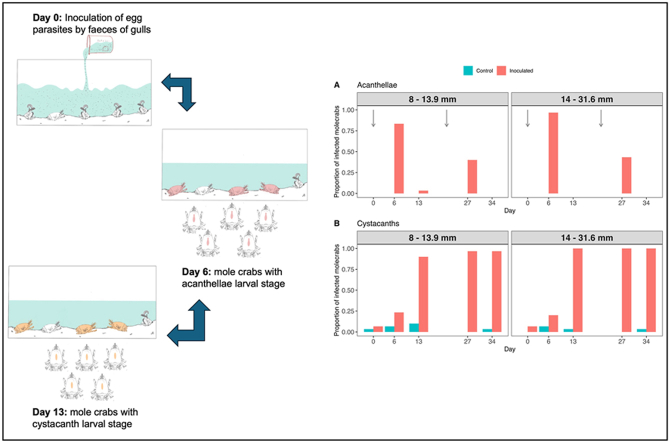

## 1 Introduction

Parasites are ubiquitous in animal populations and ecosystems, often exerting strong influence on host dynamics (Thomas et al., 2005). A fundamental pattern in parasite ecology is that parasites are heterogeneously distributed among hosts; that is, not all hosts have the same probability of becoming infected, nor do they carry the same parasite burden (Rodríguez and Valdivia, 2017). This heterogeneity is even more pronounced in parasites with complex life cycles, where smaller hosts (assuming that body size correlates with host age; Ebert, 1999) are typically less parasitized than larger ones (Rodríguez and Valdivia, 2017; Rodríguez et al., 2022). This heterogeneous distribution poses challenges for sampling and replication under natural field conditions (Cattadori et al., 2008; Filazzola and Cahill Jr, 2021). Conducting experiments in the field reduces the likelihood of obtaining replicable samples, in contrast to other biological disciplines that work under controlled conditions. Moreover, because hosts accumulate parasites throughout their ontogeny, collecting both parasitized and non-parasitized individuals across a full range of host sizes becomes difficult. Consequently, under these natural conditions it is not feasible to identify infected individuals prior to sampling, and infection must be determined retrospectively. In this context, manipulative experiments become essential, as they allow researchers to isolate and control the main factors of interest, such as host size and parasite infection, thereby enabling robust and sound hypothesis testing. Experimental infections thus provide a powerful framework to control host variability, stratify and balance sample sizes, and improve the statistical reliability of studies in complex host-parasite systems.

Various experimental approaches have been developed to study parasitized hosts under controlled conditions (Franceschi et al., 2008; Thünken et al., 2010, 2018; Dianne et al., 2011, 2012, 2014). One common strategy involves extensive field sampling across host ontogenetic stages, locations, and environmental conditions, followed by dissection to determine infection status and parasite load (Altman and Byers, 2014; Rodríguez and Valdivia, 2017; Balboa-Figueroa et al., 2019; Rodríguez et al., 2017a, 2022). Other technique involves collecting infected and uninfected individuals from historically parasite-free areas, using them as control groups for experimental treatments (Byers et al., 2008; Goedknegt et al., 2019; Díaz-Morales et al., 2022). However, these methods present two major limitations. First, individual infection status is unknown prior to experimentation, preventing true a priori hypothesis testing; secondly, it is difficult to balance the number of infected and uninfected individuals, which limits replication and statistical power. In some systems, parasites can be visually detected in transparent-bodied intermediate hosts, allowing pre-selection based on infection status (Duclos et al., 2006; García-Varela et al., 2013; Thünken et al., 2018). For example, in species with translucent cuticle, such as the amphipod *Gammarus pulex*, it is possible to detect acanthocephalan cystacanths (i.e. fully developed infective larval stage) through direct observation (Bakker et al., 1997; Thünken et al., 2010, 2018). These visual techniques help homogenize sample groups and improve replication by allowing pre-selection based on infection status. However, such approaches are only feasible in hosts with transparent exoskeletons. In most invertebrates with opaque cuticles, infection status cannot be determined non-lethally prior to dissection (Balboa-Figueroa et al., 2019; Rodríguez et al., 2017a, 2017b, 2022). Furthermore, in species where parasites reside within internal body cavities such as the coelom, there are currently no effective antiparasitic treatments or non-lethal methods to clear infections before experiments. In other systems, intermediate hosts have been artificially infected by directly inoculating parasites under the cuticle or carapace (Pulgar et al., 1995). While effective in inducing infection, these methods bypass the natural transmission route and may not accurately reflect ecological conditions. Therefore, developing methodologies that allow for controlled, ecologically relevant infection remains a key challenge in experimental parasitology.

Existing methodologies manipulate parasite prevalence and burden experimentally in intermediate hosts, often aiming to balance sample sizes across treatments (Mouritsen, 2002; Koprivnikar and Poulin, 2009; Larsen and Mouritsen, 2014; Díaz-Morales et al., 2022). Dianne et al. (2012) extracted parasite eggs from mature females in definitive hosts’ intestines and deposited them on intermediate hosts’ food, increasing infection probability compared to placing eggs directly in the habitat (water). However, this requires separating nearly 100 eggs per host to ensure acceptable infection success (Dianne et al., 2011, 2014). Another method involves screening hosts for parasites and inducing parasite emergence, mainly cercariae, by manipulating factors like light, temperature, and salinity (Mouritsen, 2002; Poulin, 2006; Larsen and Mouritsen, 2014). Subsequently, infected and potentially uninfected hosts are kept separately and re-infected before experiments (Koprivnikar and Poulin, 2009; Díaz-Morales et al., 2022). Nevertheless, these techniques cannot guarantee that initial deworming was successful or that reinfection results solely from experimental inoculations rather than natural infection. In parasites with complex life cycles, definitive hosts release infective stages through faeces, which intermediate hosts consume. Therefore, inoculations using faeces containing infective stages could enable better sample balancing and replication.

The use of faeces to detect enteroparasite infections is common in human, veterinary, and wildlife medicine (Mas-Coma et al., 2014; Wolf et al., 2014; De Nys et al., 2015; Rodríguez and George-Nascimento, 2021). While faecal samples are widely used for diagnosis and, more recently, for treatments such as faecal microbiota transplantation in humans (Messias et al., 2018; Lin et al., 2020), their application as a tool for experimental infections in ecological studies remains scarce (Aznar et al., 2012; Rodríguez and George-Nascimento, 2021). In parasites with complex life cycles, definitive hosts release infective stages through faeces, which are then ingested by intermediate hosts. Using faeces for experimental inoculations allows researchers to generate a priori hypotheses, control sample sizes, and obtain sufficient replication in ecological studies of parasitism.

Here, we propose a novel, reliable experimental methodology to inoculate faeces containing parasite eggs to intermediate hosts. We apply this methodology in a complex parasite system that includes the acanthocephalan *Profilicollis altmani*, crustaceans, the mole crab *Emerita analoga* of different body sizes, and the faeces of one of its definitive seagull hosts, the grey gull *Leucophaeus modestus*. This study had three main aims: (i) to infect intermediate hosts of different ontogenetic stages, under controlled conditions using faecal samples with parasite eggs; (ii) to generate a post-inoculation increase of parasite burden in hosts regardless of body size; and (iii) demonstrate the ability to balance infected and uninfected hosts across size classes, enabling a priori hypotheses and improved replication relative to natural field conditions.

## 2 Materials and methods

### 2.1 Host collection and acclimatization

Mole crabs (*Emerita analoga*) were collected from August 2018 to November 2019 at Curiñanco, southern-central Chile (39°44′07"S – 73°23′23"W), following the natural recruitment periods described for the region (Contreras et al., 1999). Crabs were collected during low tide using plastic corers (0.03 m^2^), buried to a depth of approximately 30 cm. Juvenile crabs visually estimated to be smaller than 9 mm were collected in the field, and in the laboratory, individuals were measured and only those < 9 mm were maintained for experiments. Crabs collected during the first, second, and third events (August 2018, December 2018, and March 2019) were assigned to Group 1, and reared for 9–16 months, reaching final cephalothorax lengths of 14–31.6 mm (large crabs). Group 2 was formed by crabs collected during the fourth, fifth, and sixth events (June 2019, September 2019, and November 2019), which were also < 9 mm at collection and grew to 8–13.9 mm (small crabs) over 3–10 weeks. The apparent discrepancy in minimum size between groups arises because both included only crabs smaller than 9 mm; in Group 2, the minimum recorded size was 8 mm, corresponding to the smallest individuals collected and maintained in the laboratory. Multiple collection periods were necessary to coincide with peak recruitment of juveniles and to compensate for laboratory mortality, ensuring enough healthy crabs for the experiments. Across both groups, median and maximum cephalothorax length were 13.9 mm and 31.6 mm, respectively. A total of 350 individuals were captured per sampling event, transported alive to the Coastal Ecology Laboratory of Universidad Austral de Chile in aerated buckets of natural seawater during drives of less than 1 h.

In the laboratory, crabs were maintained in 60 × 30 × 25 cm aquaria. The bottom (*c.*1/3 of aquarium height) was filled with field-collected sand that had been thoroughly washed prior to use. The sand was first rinsed with tap water and subsequently washed several times with distilled water until the rinse water remained clear, in order to remove debris and any potentially contaminating particles. Each aquarium was filled with filtered seawater (1 μm and 5 μm filters, plus a carbon and UV-C (200–280 nm) filter), and kept with constant aeration and at 15 °C. The mole crabs were fed daily with 150 ml of *Isochrysis galbana* microalgae, which were grown in Microalgae Laboratory of Universidad Austral de Chile, following the protocol of Guillard (1975). The water in each aquarium was replaced once a week. In this way, the crabs grew under cultivated conditions without external contaminant agents and neither the possibility of naturally parasitizing. Large mole crabs were acclimatized and maintained in these aquaria under bubbling seawater and ad libitum feeding conditions for 9 to 12 months. Similarly, small mole crabs were acclimatized under the same conditions, with circulating seawater and ad libitum feeding, but for a shorter period of 3 to 10 weeks. Prior to infection experiments, each size group (large and small mole crabs) was divided into four aquaria: two were inoculated with acanthocephalan parasite eggs and microalgae, and two served as controls (microalgae only). Each aquarium contained approximately 250–300 individuals.

### 2.2 Faeces collection and egg detection

**Fig. 1:**
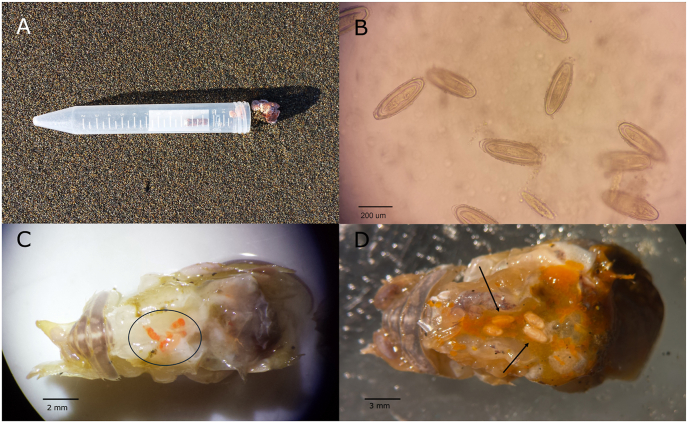
**A** Faecal sample from a grey gull (*Leucophaeus modestus*) in Curiñanco beach, Chile. **B** Eggs of *Profilicollis altmani* extracted from faeces of the grey gull. **C** Acanthellae of *P. altmani* in the hemocoel of the mole crabs *Emerita analoga* (cephalotorax removed). Circle indicates enclosed acanthellae. **D** Cystacanths of *P. altmani* in the hemocoel of the mole crab, *E. analoga* (cephalotorax removed); arrows indicate cystacanths.

During December 2019, at Curiñanco Beach, 15 fresh faecal samples (approximately 3–5 ml) from the grey gull *Leucophaeus modestus* were collected (Fig. 1A). Using a telescope, individuals actively foraging along the shoreline were kept in view, that is, visually followed until defecation occurred. Faeces were stored in 50 ml sterilized Falcon tubes and transported to the Coastal Ecology Laboratory in a cooler to maintain freshness.

The eggs of *P. altmani* in the faeces were detected using a microscope (4× ; Presswell and Lagrue, 2016). The identification was based on previous work by Smith and Rodríguez (2025), who described and photographed the eggs of this species in detail. The species identity was initially confirmed in Rodríguez et al. (2017a), where adult *P. altmani* were recovered from the intestines of *L. modestus* and genetically verified, providing a reliable reference for the egg morphology. In our samples, the eggs appeared orange, elongate-oval, and were consistent with these published descriptions. From each faecal sample, three small portions were extracted and diluted in 20 ml of sterilized marine water each. Each portion was analysed to corroborate the presence of parasite eggs (Fig. 1B).

### 2.3 Infection procedure

**Fig. 2:**
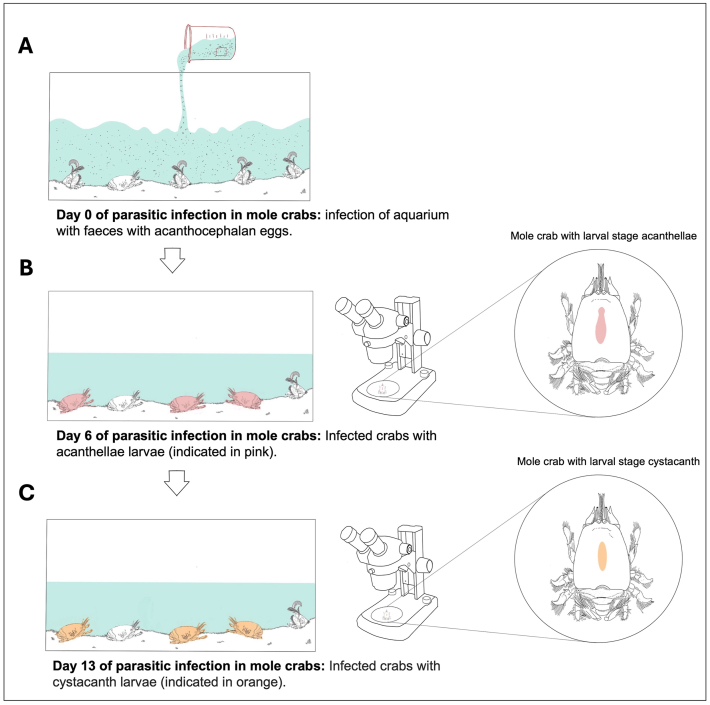
Experimental methodology of egg inoculation. **A** Inoculation of acanthocephalan *Profilicollis altmani* eggs obtained from seagull’s faeces. **B** Detection of the acanthella larval stage in the crustacean *Emerita analoga* from 6 days after inoculation*.***C** Detection of the cystacanth larval stage in the crustacean *Emerita analoga* from 13 days after inoculation.

Prior to infection, all mole crabs were deprived of food for 24 h. Aquaria designated for infection were inoculated with 1.8 g of faeces diluted in 0.3 L of filtered seawater, using whole faeces without separating solid from liquid. For each inoculation event, fresh faeces were collected following the same procedure as described above. Control aquaria were inoculated with the same amount of cleaned sand and filtered seawater. After 24 h, the water was replaced in all aquaria. Six days after inoculation, 30 mole crabs from each aquarium were dissected and inspected under a binocular microscope to detect and count acanthocephalan larvae (acanthellae and cystacanths) (Fig. 1C–D, Fig. 2). Dissections were repeated on Day 13. On Day 21, a second inoculation was performed with the same quantity of faeces (1.8 g in 0.3 L), following the initial protocol (Fig. 2). Thirty days after the first inoculation, 30 mole crabs from each aquarium were dissected and inspected again, with a final dissection on Day 34. During each dissection, the cephalothorax length (mm) of each mole crab was measured to account for growth during the acclimatization period. The presence and number of acanthellae and cystacanths per host were recorded (Fig. 1C–D, Fig. 2). Parasitological descriptors were calculated following Bush et al. (1997) and Rózsa et al. (2000). We used cystacanth occurrence (either presence or absence) and abundance (number of individuals per host) in the formal statistical analyses.

### 2.4 Statistical analyses

We used information theory to assess the effects of parasite inoculation, time, host’s body size, and their interactions on parasitosis (Burnham et al., 2011). The candidate models included both separate and interactive effects on parasitosis (Table 1). Cystacanth occurrence and abundance were analysed with binomial (logit link) and Poisson (log link) generalised linear models, respectively. Model parameters were estimated through maximum likelihood. Bias-corrected Akaike Information Criterion (AICc) was used to select the model with the highest empirical support. Model selection was based on Δ_i_, expressed a AICc_i_ - AICc_min_, Akaike weight, which represents the probability of each model as $w_{i}=Prob\{model\;g_{i}\;|\;data\}=\frac{l_{i}}{\sum_{j=1}^{R}l_{j}}$, where $l_{i}=L\left(g_{i}\;|data\right)=e^{-0.5\Delta_{i}}$. The evidence ratio of the best-supported model was calculated as W_top_/W_j_ (Burnham et al., 2011). A likelihood ratio-based pseudo-coefficient of determination (*R*^2^) was calculated for each top model.

**Table 1:** Model set used to evaluate the effects of parasite inoculation, time, and host body size on infection status and intensity. The models vary in complexity, including additive and interaction terms among inoculation treatment, time post-inoculation, and host size. The notation column indicates the explanatory variables included in each model: “+” indicates additive effects, and “∗” indicates interactions.

Model ID	Description	Notation
G1	Parasite inoculation	$\beta_{0}+\beta_{1}X_{1}$
G2	Inoculation + time	$\beta_{0}+\beta_{1}X_{1}+\beta_{2}X_{2}$
G3	Inoculation + size	$\beta_{0}+\beta_{1}X_{1}+\beta_{3}X_{3}$
G4	Inoculation + size + time	$\beta_{0}+\beta_{1}X_{1}+\beta_{2}X_{2}+\beta_{3}X_{3}$
G5	Inoculation + time + inoculation ∗ time	$\beta_{0}+\beta_{1}X_{1}+\beta_{2}X_{2}+\beta_{1,2}\left(X_{1}*X_{2}\right)$
G6	Inoculation + size + inoculation ∗ size	$\beta_{0}+\beta_{1}X_{1}+\beta_{3}X_{3}+\beta_{1,3}\left(X_{1}*X_{3}\right)$
G7	Inoculation + time + size + inoculation ∗ time ∗ size	$\beta_{0}+\beta_{1}X_{1}+\beta_{3}X_{3}+\beta_{1,2,3}\left(X_{1}*{X_{2}*X}_{3}\right)$
G8	Intercept only	$\beta_{0}$

Since acanthellae are still in early development and unable to infect the definitive host, they were excluded from the statistical analysis (Balboa-Figueroa et al., 2019).

All statistical analyses were undertaken in R 4.0.1 (R Core Team, 2020). Linear mixed-effects models were computed with the package *lme4* (Bates et al., 2015).

## 3 Results

### 3.1 Larval development: Transition from acanthellae to cystacanths across two inoculation events

**Fig. 3:**
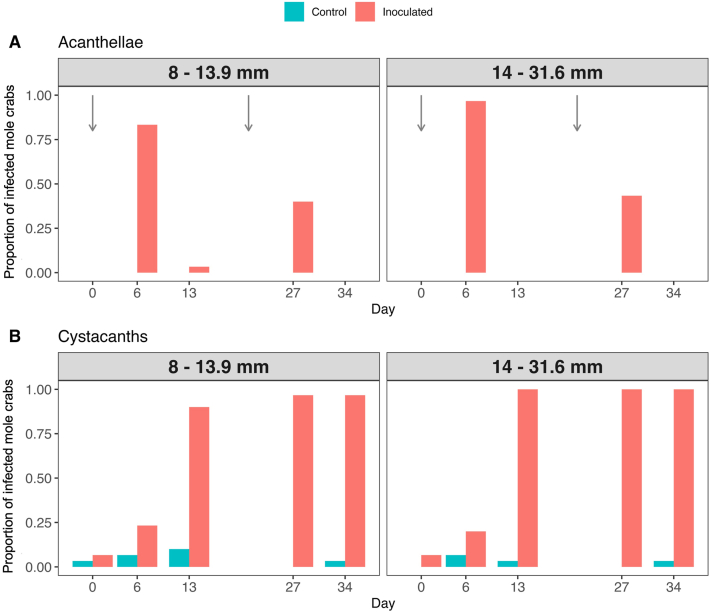
Proportion of infected mole crabs by acanthocephalan parasites during the egg inoculation experiment in two host body size groups over the course of the experiment. **A** Proportion of infected mole crabs by acanthellae following egg inoculation. **B** Proportion of infected mole crabs by cystacanths following egg inoculation. Arrows indicate the day on which parasite egg inoculation was performed. The first inoculation took place on Day 0 and the second on Day 21. On Days 0, 6, 13, 27, and 34, 30 mole crabs were examined on each sampling date.

**Fig. 4:**
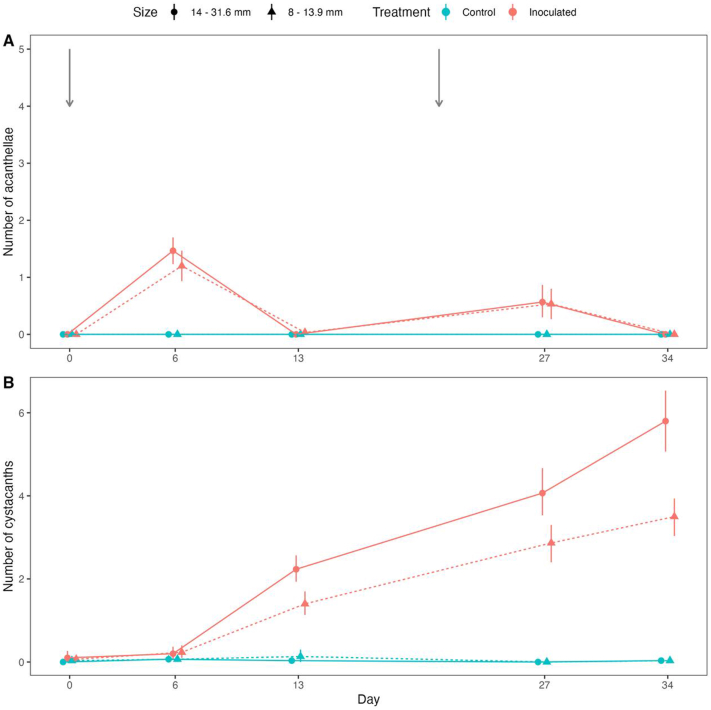
Number of acanthocephalan parasites (abundance) during the egg inoculation experiment in two host body size groups over the course of the experiment. **A** Number of acanthellae developed in *Emerita analoga* following egg inoculation. **B** Number of cystacanths developed in *E*. *analoga* following egg inoculation. Arrows indicate the day on which parasite egg inoculation was performed. The first inoculation took place on Day 0 and the second on Day 21.

Before the first inoculation, 30 mole crabs were examined; no individuals were infected with acanthellae, and only few (*n* = 5) were infected with cystacanths (Fig. 3A and B). After the first inoculation, the prevalence of acanthellae in large mole crabs was generally higher than in small crabs (Fig. 3A). In contrast, mean abundance was similar between both body-size groups (Fig. 4A and B). The acanthella stage emerged on Day 6 after inoculation of parasite eggs (Fig. 3A and 4A). Six days after inoculation, 83.3% of the small mole crabs were infected (Fig. 3A and B). Mean abundance and mean intensity were 1.2 and 1.4 acanthellae per host on Day 6 (Fig. 4A). On Day 6, the large mole crabs showed a prevalence of 96.6%, and mean abundance of 1.4 and intensity of 1.5 (Fig. 3, Fig. 4).

These acanthellae rapidly matured into the cystacanth stage (Fig. 3B). By Day 13, most acanthellae had disappeared, coinciding with a marked increase in cystacanth prevalence (Fig. 3B). Small crabs exhibited 90% prevalence, with mean abundance and intensity of 1.4 and 1.5 cystacanths per host, respectively; large crabs reached 100% prevalence with both mean abundance and intensity of 2.2 cystacanths per host, confirming successful parasite development within the hosts (Fig. 3B and 4B).

The second inoculation, taking place on Day 21, resulted in an increased parasitosis across crab’s body sizes (Fig. 4). For example, by Day 27, small crabs showed 40% prevalence of acanthellae, with mean abundance and intensity of 0.5 and 1.3 acanthellae per host, respectively (Fig. 3, Fig. 4). Large crabs exhibited 43.4% prevalence, with mean abundance and intensity of 0.56 and 1.3 acanthellae per host, respectively (Fig. 3, Fig. 4). As in the first infection cycle, these acanthellae matured rapidly: by Day 34, acanthellae were no longer observed, and cystacanths predominated (Fig. 3, Fig. 4). At this moment, small crabs reached 97.6% prevalence of cystacanths, with mean abundance and intensity of 3.5 and 3.6 cystacanths per host, respectively; large crabs reached 100% prevalence, with mean abundance and intensity both at 5.8 cystacanths per host (Fig. 3, Fig. 4).

**Fig. 5:**
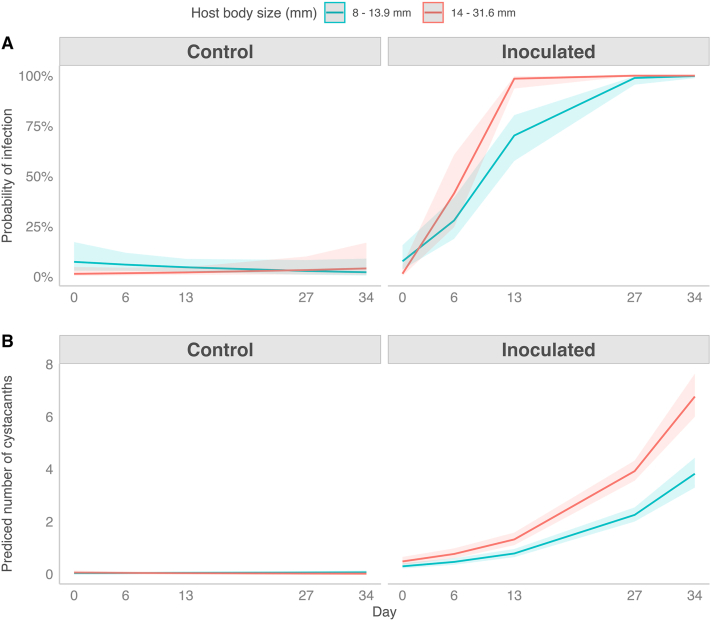
**A** Probability of cystacanth infection in mole crabs as a function of host body size, before and after parasite egg inoculation, over time. **B** Predicted number of cystacanths in mole crabs as a function of host body size, before and after parasite egg inoculation, over time.

The analysis of model selection supported the effectiveness of the experimental inoculation of parasite eggs. For the probability of occurrence of cystacanths (for brevity, referred to as infection probability hereafter), the model with the highest empirical support (lowest AICc) was that including the separate and interactive effects of inoculation, time, and host’s body size (G7 in Table 1). The probability of G7 of being the best model given the data (Akaike weight, W7) and model set was 0.97, while that of the model with the closest Δ_i_ was 0.03 (G5, Δ_5_ = 6.94; Table 2). Thus, the empirical support for model G7 was *c.*32 times that of the closest competing model (evidence ratio = 32.1). Importantly, G7 was 2.9 × 10^112^ more likely than the null model (G8 in Table 2), which provides a very strong empirical support to the time-, and body size-dependent effect of the inoculation on the probability of infection. Model G7 accounted for 81% of the variation in infection probability (pseudo-*R*^2^ = 0.81). The predicted probability of infection steeply increased over time as a result of the inoculation; this increment was faster as the host’s body length increased (Fig. 5A, Table 2).

**Table 2:** Summary of information-criteria-based model selection for the probability of infection (occurrence of cystacanths) in mole crabs. Models include effects of inoculation (I), time (T), host body size (S), and their interactions. Columns show degrees of freedom (df), corrected Akaike Information Criterion (AICc), difference in AICc relative to the best model (Δ), likelihood (L), Akaike weights (W), and evidence ratios (ER). The best-supported model (G7) includes the three-way interaction (I∗T∗S), indicating that inoculation effects on infection probability vary with time and host size.

Model ID	I	T	S	I∗T	I∗S	I∗T∗S	df	AICc	Δ	L	W	ER
G7	+	+	+			+	6	252.00	0.00	1.00 × 10°	9.70 × 10^-1^	1.00 × 10°
G5	+	+		+			4	258.94	6.94	3.11 × 10^-2^	3.02 × 10^-2^	3.21 × 10^1^
G2	+	+					3	329.07	77.07	1.84 × 10^-17^	1.79 × 10^-17^	5.45 × 10^16^
G4	+	+	+				4	329.95	77.94	1.19 × 10^-17^	1.15 × 10^-17^	8.42 × 10^16^
G3	+		+				3	490.29	238.29	1.81 × 10^-52^	1.75 × 10^-52^	5.54 × 10^51^
G1	+						2	490.39	238.39	1.72 × 10^-52^	1.66 × 10^-52^	5.83 × 10^51^
G6	+		+		+		4	491.44	239.44	1.02 × 10^-52^	9.85 × 10^-53^	9.84 × 10^51^
G8							1	769.92	517.91	3.44 × 10^-113^	3.34 × 10^-113^	2.91 × 10^112^

The number of cystacanths per host, i.e. abundance, systematically responded to the experimental inoculation over time. The model including the separate and interactive effects of inoculation, time, and host’s body size, scored the best empirical support, evidenced by the lowest AICc (G7 in Table 3). The Akaike weight of G7 was 0.99, and this model was 166 times more likely than the closest competing model (G4, Δ_4_ = 10.22, W4 = 0.006, evidence ratio of G7 *vs* G4 = 165.7). The empirical evidence for G7 was 3.34 × 10^276^ times that of the null model (G8 in Table 3), providing very conclusive support to the effect of the inoculation on intensity. Model G8 accounted for 91% of the variation in infection probability (pseudo-*R*^2^ = 0.91). The predicted intensity steeply and nonlinearly increased over time as a result of the inoculation and this increment was faster for larger hosts (Fig. 5B,Table 4).

**Table 3:** Summary of information-criteria-based model selection for parasite intensity (number of cystacanths per host) in mole crabs. Models include the effects of inoculation (I), time (T), host body size (S), and their interactions. The table shows degrees of freedom (df), corrected Akaike Information Criterion (AICc), difference in AICc relative to the best model (Δ), likelihood (L), Akaike weights (W), and evidence ratios (ER). The best-supported model (G7) includes the three-way interaction (I∗T∗S), indicating that the intensity of infection depends on the combined effects of inoculation, time, and host size.

Model ID	I	T	S	I∗T	I∗S	I∗T∗S	df	AICc	Δ	L	W	ER
G7	+	+	+			+	6	899.36	0	0.9 × 10°	1.0 × 10°	1.0 × 10°
G4	+	+	+				4	909.58	10.22	6.0 × 10^-3^	6.0 × 10^-3^	1.7 × 10^-2^
G5	+	+		+			4	944.53	45.17	1.6 × 10^-10^	1.6 × 10^-10^	6.4 × 10^9^
G2	+	+					3	959.41	60.05	9.1 × 10^-14^	9.1 × 10^-14^	1.1 × 10^13^
G6	+		+		+		4	1358.10	458.74	2.4 × 10^-100^	2.4 × 10^-100^	4.1 × 10^99^
G3	+		+				3	1358.64	459.28	1.8 × 10^-100^	1.9 × 10^-100^	5.4 × 10^99^
G1	+						2	1425.67	526.31	5.1 × 10^-115^	5.2 × 10^-115^	1.9 × 10^114^
G8							1	2172.80	1273.44	3.0 × 10^-277^	3.0 × 10^-277^	3.3 × 10^276^

**Table 4:** Summary of best (K-L) models for infection probability and parasite intensity (cystacanths). Estimates, standard errors (SE), and *z*-values for fixed effects are shown, including inoculation treatment, time, host body size, and their interactions. These models explain how these factors influence the presence and number of cystacanth parasites in mole crabs.

	Infection probability			Intensity		
	Estimate	SE	*z*-value	Estimate	SE	*z*-value
Intercept	−0.93	0.94	−0.99	−3.86	0.54	−7.15
Inoculation	0.04	0.61	0.07	2.18	0.45	4.80
Time	−0.10	0.06	−1.58	0.07	0.01	5.99
Size	−0.15	0.06	−2.56	0.04	0.02	2.20
Control∗Time∗Size	0.01	0.00	1.57	0.00	0.00	−2.92
Inoculated∗Time∗Size	0.03	0.01	5.10	0.00	0.00	0.30

## 4 Discussion

This study demonstrated that the experimental inoculation with fresh gull faeces carrying eggs of the acanthocephalan *Profilicollis altmani* enables effective, predictable, and repeatable infections in marine hosts maintained under laboratory conditions. While experimental infection techniques are well-established in parasitology, our work illustrates the controlled application of such a method in a marine system, enabling standardized manipulation of key variables, numerical balancing of experimental groups, and simultaneous availability of infected and uninfected hosts. We successfully infected mole crabs (*Emerita analoga*) of different sizes, observing larval stages from Day 6 onwards and a progressive accumulation of cystacanths up to Day 34. The statistical models that best explained infection probability and abundance, therefore, included the interactive effects of treatment, time, and host body size, showing high explanatory power (pseudo-*R*^2^ > 0.80). This technique, based on administering parasite eggs naturally present in the faeces of the definitive host, emerges as a robust tool for numerically balancing experimental groups, allowing for the availability of both infected and uninfected hosts in controlled proportions. Based on these findings, we will discuss (i) the relevance of controlled infection models in parasitic ecology, (ii) the limitations of natural sampling in systems with cryptic or heterogeneous infections, (iii) the ecological value of statistically homogenizing parasite burden, and (iv) the potential of this approach for future experimentation in ecophysiology, behavior, and ecotoxicology, both in marine and other aquatic systems.

One of the major challenges in studying parasites with complex life cycles lies in obtaining naturally infected and uninfected hosts across their entire ontogenetic range. In natural populations, larger individuals often accumulate higher parasite loads, making it difficult to disentangle the effects of host size from infection outcomes (Ebert, 1999; Rodríguez and Valdivia, 2017; Lorenti et al., 2018; Rodríguez et al., 2022). Experimental infections offer a key advantage in this context because they allow researchers to directly study the causality of phenotypic traits following parasite infection. In natural infections, it is often impossible to determine whether changes in host traits such as behavior, growth, or physiology, are caused by the parasite or by pre-existing host conditions (Balboa et al., 2009). By controlling infection under laboratory conditions, researchers can generate balanced groups of infected and uninfected hosts, disentangle the effects of host age and body size from those of parasitism, and test a priori hypotheses with higher statistical reliability (Dianne et al., 2011, 2012, 2014; Thünken et al., 2010, 2018). In this experiment, only presumptively uninfected juvenile hosts (<9 mm; Rodríguez and Valdivia, 2017) were collected and reared under controlled conditions until they reached larger sizes, with the aim of eliminating this source of bias. Nevertheless, it is important to note that some crabs were naturally infected prior to collection, despite being selected according to the previously reported minimum infection size threshold (≥ 9 mm cephalothorax length) (Rodríguez and Valdivia, 2017). This experimental approach allowed us to isolate the effect of body size on infection, revealing that larger hosts not only acquired more intense infections but did so in a shorter period of time. This pattern can be explained by functional mechanisms associated with feeding. *Emerita analoga* is an active filter feeder, and larger individuals process significantly greater volumes of water and sediment than smaller conspecifics (Dugan and Hubbard, 1996; Contreras et al., 1999). Consequently, larger hosts have higher encounter rates with parasite eggs and acquire infections more rapidly and with greater intensity, whereas smaller crabs filter less water and are exposed to lower effective egg densities (Smith, 2007; Balboa et al., 2009; Rodríguez and Valdivia, 2017). By simultaneously inoculating both size classes under controlled conditions, we were able to disentangle the effects of host age from those associated with filtration activity, demonstrating that body size is a mechanistic determinant of parasite exposure. Overall, these results highlight the ecological relevance of controlling host size and infection status, while also illustrating how this system can be used to test specific hypotheses about the determinants of parasite intensity and probability in a predictable and replicable experimental framework.

The main novelty of this study lies in the use of fresh faeces from definitive hosts as a vector to induce infections in intermediate hosts. While faeces are routinely employed for parasite diagnosis (Mas-Coma et al., 2014; Presswell and Lagrue, 2016; Rodríguez and George-Nascimento, 2021), their application as an experimental tool in ecological studies is almost non-existent. This technique leverages the natural faecal-oral transmission route of the parasite and circumvents the complex procedures involved in isolating eggs or larvae (Dianne et al., 2012; Presswell and Lagrue, 2016). Rather than presenting this method as an absolute innovation, we emphasize its practical utility in controlling experimental infections in a marine system where cystacanths cannot be visually detected. By enabling a realistic and efficient inoculation without the need for direct manipulation of the parasites, its use is facilitated especially in marine organisms with opaque cuticles, such as *Emerita analoga*, where visual detection of infection is impossible (Balboa-Figueroa et al., 2019). For example, the transparent cuticle of the amphipod *Gammarus pulex* allows one to visually detect the presence of acanthocephalan cystacanths without dissection (Bakker et al., 1997; Thünken et al., 2010, 2018). This methodology thus provides a controlled framework for generating balanced groups of infected and uninfected hosts, allowing researchers to test specific hypotheses related to host size, filtration rate, and infection dynamics. In this context, the use of faeces as an experimental vector represents an effective, standardized, and ecologically relevant alternative for inducing infections in systems with technical diagnostic limitations. Therefore, this methodology expands the possibilities to study parasitic systems where infection is cryptic and difficult to standardize.

The methodology presented here not only represents a technical innovation but also opens new avenues for the development of controlled experiments in disease ecology. In systems involving parasites with complex life cycles, where infection occurs in a cryptic and (seemly) stochastic manner, controlling the infection status of hosts has remained a persistent challenge (Balboa et al., 2009; Rodríguez et al., 2017a, 2022). Our technique allows for the standardization of initial experimental conditions by generating controlled groups of infected and uninfected individuals, ensuring treatment replicability and a balanced distribution of samples across experimental groups (Albert et al., 2010; Burgess et al., 2022). This is particularly valuable in studies aiming to assess the effects of parasites on host phenotypic traits such as behavior, metabolic rate, growth, or survival, where natural infection biases could confound result interpretation (Franceschi et al., 2008; Thünken et al., 2010; Goedknegt et al., 2019). Moreover, this technique is scalable to other marine or aquaculture systems where experimental evaluation of parasite impacts under semi-controlled or laboratory conditions is desired. Its application can extend to studies on co-infections, parasite competition, changes in host life-history traits, or parasite-mediated trophic interactions, contexts in which sample size balancing is often difficult due to the nature of individual life cycles (Balboa et al., 2009; Goedknegt et al., 2019; Thünken et al., 2019). Additionally, this approach is not limited to acanthocephalans but could be applied to other parasite groups with complex life cycles whose infective stages are also released through the faeces of definitive hosts. These include nematodes, cestodes, and digeneans, where eggs or larvae are expelled by aquatic or semi-aquatic vertebrates and subsequently ingested by intermediate hosts. Therefore, experimental inoculation *via* faeces represents an ecological, efficient, and replicable pathway to induce infections across a wide diversity of parasitic systems.

Despite its advantages, the faecal inoculation method presented here has inherent limitations that should be acknowledged. First, the use of fresh faeces as the source of parasite eggs introduces variability in the exact number and viability of eggs ingested by each host, potentially leading to uncontrolled variation in infection doses among individuals (Dianne et al., 2011). This contrasts with methods using isolated and quantified parasite stages, which allow for precise dosing. Additionally, faecal suspensions may contain a mixture of parasite species and developmental stages, which could confound interpretations if the parasite community is diverse (Mas-Coma et al., 2014). Moreover, environmental factors affecting egg viability and infectivity within the faecal material, such as temperature or microbial activity, could influence infection success and are challenging to standardize fully (Presswell and Lagrue, 2016; Hopkins et al., 2022). Lastly, although the method mimics natural transmission routes, it does not fully replicate the complexity of environmental exposure *in situ*, such as spatial heterogeneity of eggs, host behavior, and ecological interactions that may influence infection dynamics in the wild (Ebert, 1999; Balboa et al., 2009). Therefore, while this technique provides a robust framework for controlled experimental infections, careful consideration of these limitations is essential when extrapolating laboratory findings to natural systems.

## 5 Conclusions

This study proposes and validates a simple, effective, and ecologically realistic experimental tool for inducing parasitic infections in intermediate hosts through the inoculation of fresh faeces containing viable parasite eggs. Using *Emerita analoga* as the model host and the acanthocephalan *Profilicollis altmani* as the parasite, we demonstrated that larger hosts acquire higher infection intensities, likely due to greater filtration capacity and cumulative larval exposure over time. This technique follows the parasite’s natural faecal-oral transmission route, avoids direct manipulation of infectious propagules, and provides substantial advantages in replicability, experimental control, and applicability to systems with cryptic or hard-to-detect infections. Although the exact number of eggs per host cannot be precisely controlled, the approach maintains ecological realism and can be applied to other host-parasite systems with similar transmission strategies. Taken together, this methodology represents a significant advance for experimental ecological studies, enabling a priori hypothesis formulation, standardized infection protocols, and improved statistical inference for understanding parasite effects on host life-history traits, behavior, and population dynamics.

## Ethical approval

Not applicable.

## CRediT authorship contribution statement

**Sara M. Rodríguez:** Conceptualization, Supervision, Visualization, Writing - original draft. **Katherine Burgos-Andrade:** Methodology, Writing - review & editing. **Valentina Escares-Aguilera:** Methodology, Writing - review & editing. **Bárbara Gutiérrez:** Methodology, Writing - review & editing. **Nelson Valdivia:** Conceptualization, Data curation, Visualization, Writing - original draft.

## Funding

This work was funded by 10.13039/501100020884ANID Chile , through Fondecyt Postdoctoral grant #3190348, 10.13039/501100020884SIA-ANID grant #85220111 and 10.13039/501100002850FONDECYT grant #11250652 and #1230286.

## Declaration of competing interests

The authors declare that they have no known competing financial interests or personal relationships that could have appeared to influence the work reported in this paper.

## Data Availability

All data generated in this study are available *via* the Figshare repository at https://doi.org/10.6084/m9.figshare.31446232.
